# Circadian rhythms and diurnal patterns in the feed intake behaviour of growing-finishing pigs

**DOI:** 10.1038/s41598-023-42612-1

**Published:** 2023-09-25

**Authors:** Jacinta D. Bus, Iris J. M. M. Boumans, Jasper Engel, Dennis E. te Beest, Laura E. Webb, Eddie A. M. Bokkers

**Affiliations:** 1 Animal Production Systems Group, Wageningen University & Research, PO Box 338, 6700AH Wageningen, The Netherlands; 2 Biometris, Wageningen University & Research, PO Box 16, 6700AA Wageningen, The Netherlands

**Keywords:** Animal behaviour, Animal physiology

## Abstract

The feeding behaviour of growing-finishing pigs is an important indicator of performance, health and welfare, but this use is limited by its large, poorly-understood variation. We explored the variation in basal feed intake of individual pigs by detecting circadian rhythms, extracting features of diurnal patterns and assessing consistency over time, from day-to-day and across age. Hourly feed intake data of individual pigs (n = 110) was obtained during one growing-finishing phase, using electronic feeding stations. We applied wavelet analysis to assess rhythms and a hurdle generalised additive model to extract features of diurnal patterns. We found that circadian rhythms could be detected during 58 ± 3% (mean ± standard error) of days in the growing-finishing phase (range 0–100%), predominantly at older ages. Although the group diurnal intake pattern was alternans (small morning peak, larger afternoon peak), individual pigs showed a range of diurnal patterns that changed with age, differing mostly in the extent of night fasting and day-to-day consistency. Our results suggest that the type, day-to-day consistency and age development of diurnal patterns in feed intake show general group patterns but also differ between pigs. Using this knowledge, promising features may be selected to compare against production, health and welfare parameters.

## Introduction

The feeding behaviour of growing-finishing pigs (*Sus scrofa domesticus*, onwards referred to as ‘pigs’) can function as an important indicator for their performance^1^, health^2,3^ and welfare^4,5^. Nevertheless, before this potential can be utilised, feeding behaviour must first be well-understood at a basal level, so that normal pig-to-pig and day-to-day variation can be separated from welfare-indicative variation^6^. In pigs, feeding behaviour can be measured continuously using technologies such as camera vision^e.g.^^7,8^, radio-frequency identification (RFID) systems at the feed trough^e.g.^^9,10^, or electronic feeding stations (EFSs)^e.g.^^11,12^. EFSs combine an RFID system with a load cell or portioned filling system to record the timing of each feeding visit and its corresponding feed intake. The main variables expressing feeding behaviour are feed intake, feeding duration, feeding frequency (nutritive or non-nutritive) and feeding rate, where variables can be expressed per visit/meal or, by summing or averaging visits or meals, per hour, day, week, etc.^13,14^. Many studies have reported relationships between these variables and welfare issues^6^ (welfare is defined here as the balance between positive and negative affective experiences^15,16^), for example a reduction in intake and duration following bacterial and viral infections^17,18^ or heat stress^19^, and a reduction in intake and frequency during tail-biting outbreaks^3,5^. Based on these relationships, several authors have attempted to develop models that could detect deviations from basal feeding behaviour^20,21^, sometimes linking periods of deviation to periods of disease^22,23^. Although useful, the reliability of such detection methods should be improved further, as currently only up to 58% of health issues could be detected^23^ and 55–71% of detected issues did not have an identifiable source (i.e. were considered false)^22,23^. For tail biting specifically, machine learning methods were applied to detect up to 94% of treated tail wounds on one farm (method: k-nearest neighbours), however, when the same authors trained and tested their models on a different farm at most 50% of treated tail wounds could be detected (method: random forests)^24^. A potential path to improve model performance is to dig into the large reported variation in pigs’ basal feeding patterns^e.g.^^25,26^, as this large variation is not yet well-understood but can reduce the efficacy of prediction and deviation detection models. If we understand which aspects of basal feeding behaviour are more or less variable at the individual level, this will provide opportunities for selecting low-varying features from which more subtle abnormal (e.g. health- or welfare-induced) variation could be isolated.

One interesting and underexplored aspect of variation can be found in the circadian patterns of feeding behaviour. In this paper, circadian patterns are described along two separate dimensions, termed ‘circadian rhythms’ and ‘diurnal patterns’. Circadian rhythms refer solely to the recurrence of the behaviour along a 24 h cycle, regardless of the structure of the repeating behaviour within those 24 h. This structure is instead described by the diurnal pattern, which thus describes the type of pattern that repeats within the circadian rhythm (e.g. the timing of lower and higher quantities of feed intake). It is generally accepted that animals show circadian activity rhythms, with both physiological and behavioural activity peaks at certain times of the day. The main pacemaker of the approximate 24 h-rhythm is the suprachiasmatic nucleus, located in the hypothalamus, which sends signals to peripheral clocks situated in cells throughout the body^27^. To retain the rhythm at exactly 24 h, environmental cues (*Zeitgebers*) are required, such as the light cycle or feeding times^28^. Averaged across pigs and over time, pigs show an alternans activity pattern, which consists of a small feeding peak in the morning and a larger peak in the afternoon^29,30^. Some studies have reported that diurnal patterns can be adapted to external or internal stressors. For example, pigs shift their feeding behaviour towards night hours during heat stress^19,31^, and during social stress pigs spread out their feeding throughout day and night rather than concentrate it during the day^32,33^. In addition, it could be theorised that sick animals may adapt their diurnal pattern away from peak hours when unable to compete for access to the feeder. A recent study in dairy cows demonstrated that events of disease, stress and reproduction could be detected using changes in the circadian rhythm^34^. Nevertheless, to be able to relate certain circadian patterns to welfare issues in pigs as well, we must first know whether pigs consistently show individual diurnal patterns and, if so, what these patterns look like. Indeed, the presence of an alternans feeding pattern at group level, averaged across days, does not mean that individual pigs show this diurnal pattern on individual days, as accumulations or averages of diurnal patterns do not necessarily provide a proper reflection of the individual patterns they were based on. For example, if a pig shows a high feeding activity at 10:00 h on one day but not on the next, the average pattern will show an intermediate activity on that hour and thus reflect neither of the individual days. Consequently, whether the alternans pattern is shown by all individuals, or even on individual pig days, is currently unknown.

It has been suggested that circadian rhythms are influenced by age, with younger pigs showing less established circadian rhythms than adults^35^. During the growing-finishing phase, pigs develop from about 8–10 to 20–26 weeks of age—growing about 100 kg in this period—and change their day-level feeding behaviour as they age^6^. Although the development of circadian rhythms has been little studied in pigs, from studies in other mammals it is well-known that at birth most physiological and behavioural processes follow an ultradian (i.e. activity repeats more frequently than every 24 h) rather than a circadian rhythm^27^, with the circadian rhythm appearing gradually throughout the first year of life (e.g. after about 2–3 months in human babies^36,37^). In pigs, there is some evidence for the emergence of circadian rhythms in physiology (salivary cortisol)^38,39^ and behaviour (motor, feeding and drinking activity)^40^ with age, but first emergence ranged from 10 days to 5 months of age between the studies and was determined at group level. Additionally, studies detecting repetition in behaviour generally did not report what the detected diurnal patterns looked like, thus not providing opportunities to identify specific deviations from normal diurnal patterns beyond disturbances in the general circadian rhythm. Hence, an understanding of how feeding behaviour develops with age as well as of which aspects of diurnal patterns are consistently repeated by individual pigs is required before features representing the diurnal feeding pattern can be used to detect welfare-indicative deviations.

This study’s aims are threefold. First, we aim to explore whether the feeding behaviour of individual pigs periodically repeats from day to day—i.e. has a circadian rhythm—throughout the growing-finishing phase. Second, we aim to describe the individual diurnal patterns of pigs, in other words describe at which moments of the day feeding activity takes place. Third, we aim to see how diurnal patterns change with age, and how consistent individual pigs are in their diurnal feeding activity over time. Together, these efforts will give insight into the individual and temporal variation in the feeding behaviour of pigs. Moreover, it will provide us with additional variables reflecting circadian patterns (hereafter referred to as features), that can be combined with day-level variables to detect possible deviations from basal diurnal feeding behaviour which could be indicative of welfare issues.

## Methods

### Animals and housing

This study concerns an observational study on commercially-reared growing-finishing pigs. All methods complied with relevant guidelines and regulations according to German and EU legislation. As no invasive or harmful procedures were applied, approval of the experimental protocol by an institutional or licensing committee was not necessary, conform Dutch (Article 1 Wet op de Dierproeven, 2021, https://wetten.overheid.nl/BWBR0003081/2021-07-01) and German (Article 7 Tierschutzgesetz, 2022, https://www.gesetze-im-internet.de/tierschg/BJNR012770972.html) legislation. The study is reported in accordance with the ARRIVE guidelines.

This study involved one round of commercial growing-finishing pigs reared between December 2020 and March 2021 at a Topigs Norsvin (pig breeding company, Helvoirt, the Netherlands) fattening farm in Germany. Piétrain × (Landrace × Large White) piglets arrived at approximately 10 w of age, at a body weight of 27.5 ± 2.9 kg (mean ± stdev), and were monitored for 83 days until the heaviest pigs were transported to the slaughterhouse (8 days after all pigs were weighed at 107 ± 8.7 kg). Pigs were housed in 10 pens with 11 pigs per pen (n = 110 pigs, 1.03 m^2^/pig), spread across five rooms. In each room, one pen contained barrows and the other gilts. Each pen was equipped with fully slatted floors, two drinking nipples with ad libitum access to water, and one IVOG® EFS (Hokofarm group, the Netherlands). From the EFS, pigs could obtain one of three types of pelleted feed ad libitum (until d33, Select Delta 2: 16.2% crude protein (CP) and 13.2 MJ/kg metabolisable energy (ME); between d33–64, Select Delta 4: 15.3% CP and 13.1 MJ/kg ME; post d64, Select Delta 5: 13.8% crude protein an 13.0 MJ/kg ME; all produced by Royal Agrifirm Group, the Netherlands), where feed switches were performed by mixing the feed for 2–3 days before switching completely. Pens were equipped with a combination of a hanging wooden block, chains with plastic rings and hanging ropes, all intended as enrichment (exact provision differed between pens). Temperature reduced from 25 to 22 °C across the growing-finishing phase, with a diurnal variation of 1–2 °C and a maximum difference between rooms of approximately 2 °C. There were no occurrences of uncontrolled extreme temperatures, such as heat stress. Windows provided natural lighting, with the approximate number of daylight hours increasing from 8 to 12.5 h across the experiment. No artificial lighting was provided except during human presence in the compartment. Pigs were checked on by the caretaker twice daily, and all management procedures were determined and performed by Topigs Norsvin employees. Three pigs from two pens were removed from the study and into a sick pen, where feeding behaviour could not be monitored, before reaching slaughter weight due to health issues (after d10, d49 and d75, d49 and d75 from the same pen). No pigs had received medical treatment before being moved to the sick pens.

### Data collection and processing

IVOG® EFSs were used to obtain data on the feeding behaviour of every pig. IVOG® EFSs are single-space feeders equipped with a radio-frequency identification (RFID) antenna and a load cell. The RFID antenna detected the ear transponder of a pig upon entering the feeder, thus identifying the pig by its unique transponder number. Upon entrance, the EFS recorded the time stamp and the weight of the feed in the trough (i.e. feed is always present in the trough), and these measures were recorded again when the pig exited the feeder. A small fence protected against two pigs entering the feeder simultaneously either from the side or by jumping over the feeding pig, but otherwise the feeding pig was unprotected (i.e. there was no fence or protective crate covering the top/back or sides of the feeding pig) and thus subject to competition in the pen. A metal bar on the floor prevented pigs from lying down in front of or with the head inside the feeder. The load cell of each EFS was calibrated before the pigs arrived in the barn. As EFSs occasionally record incorrect visits, a cleaning algorithm adapted from that developed by Casey et al.^41^ and Eissen et al.^42^ was used to remove putatively incorrect visit registrations. During this process, feed intake of 7.27% of all visits was removed, of which 1.61% of all visits due to complete removal of all data beyond d63 from one broken EFS and 1.71% of all visits due to removal of all visits on pig days that had many individual visits removed, to prevent introduction of bias during aggregation (for a detailed description of removal steps, see “section 1” of the Supplementary Methods). Using the remaining visits, visit intakes were aggregated to obtain the feed intake of each pig for every hour of the growing-finishing phase (kg, sum of visit intakes). Besides feed intake, we also explored diurnal patterns in feeding duration and frequency, but as these provided similar results we only report on diurnal patterns in feed intake in this paper. Feed intake has the advantage of a relatively low range of variation, providing more easily interpretable results, and is independent of the application of a meal criterion to accumulate feeding visits into meals^13^—hence no meal criterion had to be applied in this study.

As our aim was to study basal feeding patterns, we wanted to remove pig feeding days that could be expected to deviate from basal. To this end, we removed (1) days surrounding disease (± 3 days of high disease score), as it is known that diseases can strongly affect pig feeding behaviour^2,3,22^; (2) all days before the first health observations to avoid the period of habituation to the farm and inclusion of yet unidentified sick pigs; and (3) all days after the first batch of pigs was sent to slaughter, because this causes social disturbance in the pigs left behind^43^. Diseased individuals were identified during twice weekly health observations using the health protocol presented in Bus et al.^44^. This process removed 28.4% of pig feeding days from the final dataset (details are provided in section 2 of the Supplementary Methods). The final dataset used for analysis consisted of 6348 complete days on 98 pigs from 9 pens, with a median of 65 days per pig (range 16–79 days).

### Data analysis

All data processing and analyses were performed in R, version 4.1.2^45^. Plots were created using the *ggplot2* package^46^ and tables were created using the *flextable* package^47^.

#### Detecting circadian rhythms

Wavelet analysis was applied on the hourly intake data of each pig to detect possible circadian rhythms in their feeding behaviour. Wavelet analysis is capable of detecting repetitions at a range of different frequencies (e.g. repeating every 12 h, 18 h or 24 h), located at different moments in the time series (e.g. at the beginning or at the end of the growing-finishing phase)^48,49^. It has been used for similar purposes in previous studies to detect, among others, activity rhythms in mice^50^ and polar bears^51^, but has not yet been applied to livestock species. In essence, wavelet analysis starts with a base wavelet, which is a rhythmic signal with a particular periodicity that reflects a pre-chosen form of (behavioural) repetition. This base wavelet is then translated across the time series and each time the similarity between the wavelet and the time series is calculated, a parameter referred to as “power”. By using an array of wavelets with different periods, it is possible to scan for multiple periodicities across the time series. A higher power suggests repeating behaviour of the pre-defined periodicity around that point in the time series. By comparing the power of the studied time series with the power of a simulated time series at a certain periodicity and time point, it can be tested whether the behavioural repetition is significantly stronger than random variation.

In this study, wavelet analysis was performed using the R package *WaveletComp*^52^. We used the Morlet base wavelet and a continuous wavelet transform, after previous studies on animal behavioural rhythms^49,50,53^. Behavioural repetition on all frequencies between 8 and 48 h was tested, but as no persistent periodicities were detected at any frequency besides ± 24 h (onwards referred to as a circadian rhythm), other frequencies were not further considered. Rough indications of significance values were calculated by comparing to wavelet powers for 1000 time series simulations of white noise, which would reflect random, non-repeating behaviour. Previous studies have shown that non-constant mean and variance across the time series can hamper wavelet analysis, especially in significance testing^53^, therefore hourly intake data was de-trended and corrected for amplitude changes for each pig separately. De-trending corrected for the increase in average feed intake across the growing-finishing phase. A local regression model (LOESS) was fitted to the hourly intake of every pig using the *loess()* function^45^ (span = 0.75) and de-trended data was obtained by subtracting the fit from the observed hourly feed intake. Amplitude changes occurred for example when an older pig ate during few hours of the day but had the same de-trended daily intake as a young pig that ate every hour, creating a larger amplitude in the older than the younger pig. We corrected for amplitude changes by extracting the difference between the highest and lowest hourly intake for every 7 days and dividing each data point within the 7d-window by this difference. Pig-level visualisations of the data over time were used to assess whether constant mean and variance across the time series were obtained after de-trending and amplitude correction. Finally, as wavelet analysis cannot be performed on incomplete datasets, all missing data points were replaced with zero. This was preferred over imputing missing data points as we did not want to introduce artificial structure into the data. A trial with simulated data showed that the high occurrence of zeros did not disturb the detection of repetitions in our range of interesting frequencies (around 24 h), although it could cause artefacts of moderate to high power in low frequency repetitions (< 12 h, results not shown). The wavelet power spectrum was therefore mainly interpreted for those time windows where no missing data points had been replaced.

From the results of the wavelet analysis, for each pig its wavelet spectrum was visualised, showing the power and its significance (calculated by comparing to 1000 time series simulations of white noise) of periodicities in intake across the growing-finishing phase. To obtain an approximate quantification per period of time, we extracted for each non-missing day whether a significant circadian rhythm could be detected. We calculated the median of all *P*-values of the power on the 24 time points (hourly intake) of the day between frequencies 23.5 to 24.5 h, and noted a day as showing a circadian rhythm if this median *P*-value was smaller than 0.05. For each month of the fattening phase, the proportion of days with a circadian rhythm was calculated by dividing the number of days with a median *P*-value smaller than 0.05 by the total number of available days for that pig, and this was summarised at group level using probability density. Further insight into the circadian rhythms of pigs is provided using visualisations (wavelet spectra) of five example pigs, selected for extremity in both the presence or absence of a strong circadian rhythm and other characteristics of their diurnal pattern, the latter of which is explained in the next section.

#### Describing diurnal patterns

**Figure 1:**
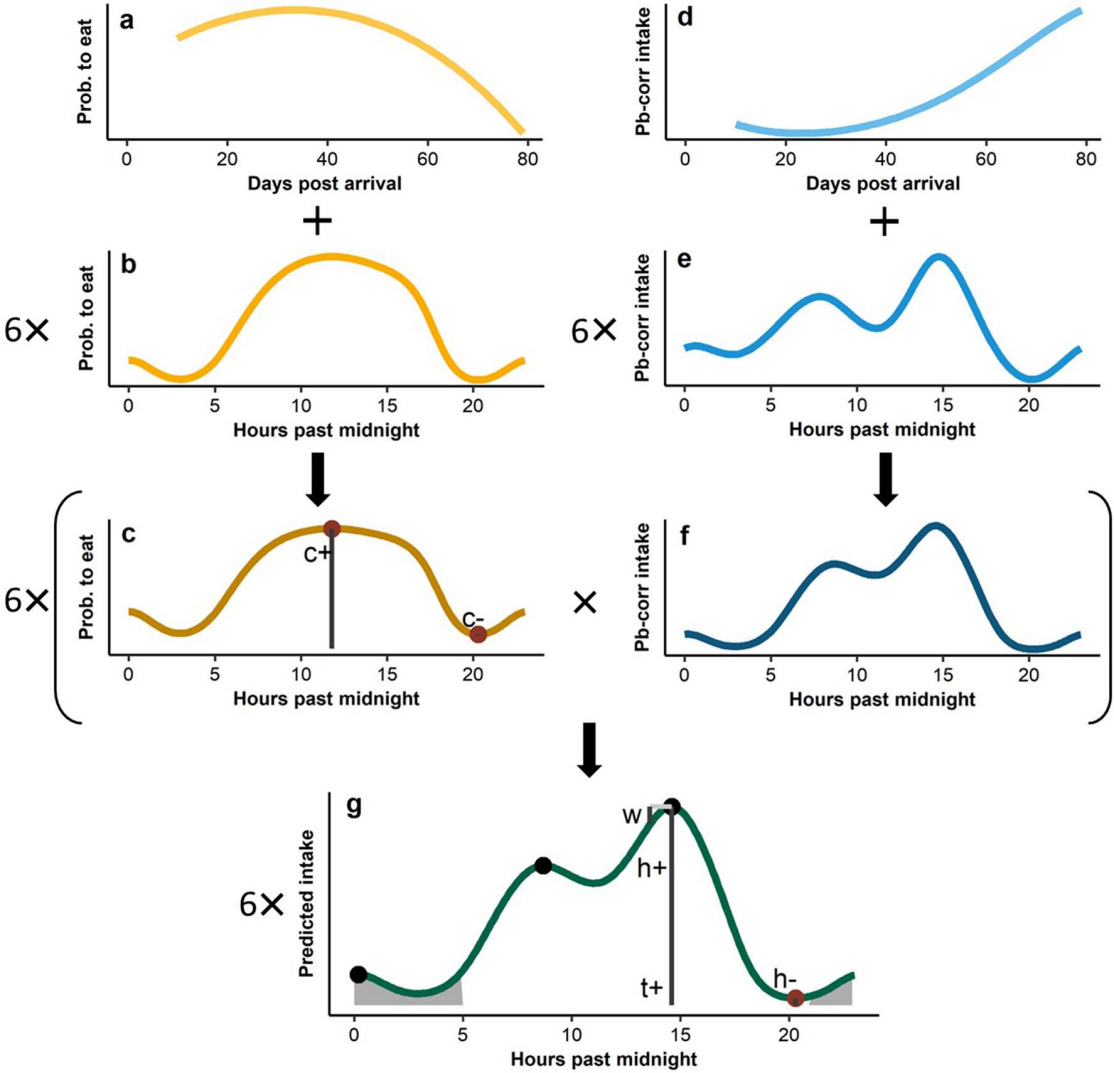
A schematic overview of the generalised additive models (GAMs) using one example pig and 14 days-period. At the top left, the probability (prob.) that a pig eats at every moment (panel **c**) is modelled by adding the probability to start eating across days (panel **a**) and the diurnal probability surrounding this daily trend for each of the six periods (panel **b**). On the right, the probability-corrected (pb-corr) intake of a pig (panel **f**) is modelled similarly, by adding the trend (panel **d**) and diurnal intake surrounding the trend of each of the six periods (panel **e**). The resulting probabilities to eat (panel **c**) and probability-correct intakes (panel **f**) are multiplied to obtain the final predicted intake of the six periods (panel **g**). Six features of diurnal patterns in feed intake were extracted from this final prediction: the height (h+), width (w), and timing (t+) of the highest peak, the height of the lowest intake (h−), the number of peaks (black dots), and the proportion of intake obtained at night (grey shading). From the hourly probability plot (panel **c**) the height of the lowest (c−) and highest (c+) probabilities of eating were extracted as an approximation of day-to-day consistency. Note that quantities (height, width and proportion of intake) and probabilities (lowest and highest) were corrected for the trend, to allow comparison between periods. The model in R code: *gamlss(intake* ~ *ga(*~ *s(hour, bs* = *“cp”, k* = *8, by* = *period)* + *s(day, bs* = *“ps”, k* = *4)), nu.formula* =  ~ *ga(*~ *s(hour, bs* = *“cp”, k* = *8, by* = *period)* + *s(day, bs* = *“ps”, k* = *4)), sigma.formula* =  ~ *period—1, data* = *na.omit(hourly.intake.data), family* = *ZAGA)*.

We used hurdle generalised additive models (GAMs, R package *gamlss*^54^) to model the diurnal patterns of each pig separately, enabling the extraction of quantitative features that describe the diurnal behaviour of individual pigs. The GAM modelled the overall trend in intake across the growing-finishing phase and added diurnal patterns to this as the within-day (i.e. hourly) variation surrounding the trend. Diurnal patterns were modelled for periods of 14 days (onwards referred to as a ‘pig period’), giving a total of 5 periods consisting of 14 days and 1 period consisting of 8 days. If fewer than 7 days were available for a pig period, the pig period was excluded. The high frequency of hours during which a pig did not eat required separate modelling of zeros and non-zeros. This was done with a hurdle model that first fitted the probability of a pig eating and subsequently fitted feed intake as a continuous response weighted for the probability of eating. The probability was fitted using a logistic regression with a logit link and the continuous part was modelled using a zero-adjusted gamma (i.e. ZAGA in *gamlss*) with a log link. Both parts of the hurdle model contained the overall trend across days (using a spline with 4 knots) and the diurnal pattern per period (using a cyclical spline with 8 knots). A visualisation of the working of the model is provided in Fig. 1, and the model code is given in the figure caption.

Model fit was checked visually for each pig period by plotting model predictions on top of the raw data. To test if modelling diurnal patterns on top of the trend was warranted, we compared the GAM with one trend and six diurnal patterns to reduced models fitting (1) only the overall trend or (2) the overall trend and only one diurnal pattern across all periods. For each pig, the full model was compared to each of these reduced models using a likelihood ratio test (function *LR.test* of the *gamlss* package^54^, *χ*^2^-test for nested models), and the percentage of pigs for which the full model had a significantly better fit compared to the reduced model (*P* < 0.05) was calculated for both of the reduced models.

From the results of the full GAM model, we extracted eight features which were calculated once per pig period. The features were selected to be representative of the range in variation in diurnal patterns observed during visualisations of the GAM results, where six described the diurnal pattern and two the day-to-day consistency in this pattern. Extracted features were: (1) the number of peaks in feed intake (count of occurrences where the model derivative was zero and values shifted from increasing to decreasing, function *findpeaks()* of R package *pracma*^55^); (2) the timing of the highest peak in hours post-midnight; (3) the height of the highest intake peak in predicted de-trended feed intake (de-trended g/h); (4) the width of the highest peak, calculated as the mean difference in height half an hour before and after the peak (de-trended g/h, higher values represent narrower peaks); (5) the height of the lowest intake (de-trended g/h); (6) the proportion of intake obtained at night, calculated as the predicted intake obtained between 21:00 and 05:00 h divided by the total predicted intake; and, for day-to-day-consistency (7) the lowest probability to start eating, extracted as the minimum probability fit of the hurdle model (rather than the intake fit); and (8) the highest probability to start eating, extracted as the maximum of the probability fit of the hurdle model. These latter probability parameters (7 and 8) approximate the proportion of days within the 14 days period that a pig was (not) eating at that time, thus a low value for the minimum probability and a high value for the maximum probability would represent a pig that eats at similar moments each day within the period. Extracted features are visualised along with the working of the model in Fig. 1c,g.

For the extracted features, it was tested whether they change across the 14d-periods of the growing-finishing phase—in other words, as pigs age. We used a linear mixed model with period as a fixed factor, pen and pig as random factors, and an AR(1) structure fitted to the residuals to model correlation between consecutive periods (R package *glmmTMB*^56^). To meet normality assumptions (tested using a histogram, QQ-plot and Shapiro–Wilk test (W ≥ 0.9) on the raw data and model residuals), features were if necessary transformed using square root (features: lowest probability of eating, lowest intake), logarithm (features: width of the highest peak, highest intake) or a fixed value minus the square root (feature: timing of the highest peak). Using these models, we tested for an effect of period using a χ^2^ test, and if significant (*P* < 0.05), a post-hoc Tukey test was used to compare the periods pairwise (R package *emmeans*^57^). Besides the general change of features across the periods, we were also interested in how consistent individual pens and pigs were from period to period. For this, we calculated the intraclass correlation coefficient (ICC) for both pens and pigs by dividing the variation explained by pen/pig effects in the model by the total variation. The total variation consisted of the variation explained by the pig effects, pen effects, and the diagonal of the autoregressive correlation matrix. ICCs were interpreted as weak (> − 0.4 and < 0.4), moderate (≤ − 0.4 and > − 0.6, or ≥ 0.4 and < 0.6) or strong (≥ 0.6 or ≤ − 0.6).

#### Associations between and within circadian rhythms and diurnal patterns

To be able to identify true diurnal patterns rather than individual features of them, the features of the circadian rhythms (wavelet analysis) and diurnal patterns (GAM features) were compared to each other. For each pig and period, the proportion of days with a circadian rhythm was calculated, corrected for missing days and with a minimum threshold of 7 available days per period. Subsequently, for each period all features of the wavelet analysis and GAMs were compared to each other using Spearman rank correlation, with the *cor()* function of the *stats* package^45^. Similar to ICC scores, correlation coefficients were interpreted as weak (> − 0.4 and < 0.4), moderate (≤ − 0.4 and > − 0.6, or ≥ 0.4 and < 0.6) or strong (≥ 0.6 or ≤ − 0.6).

## Results

### Detecting circadian rhythms

**Figure 2:**
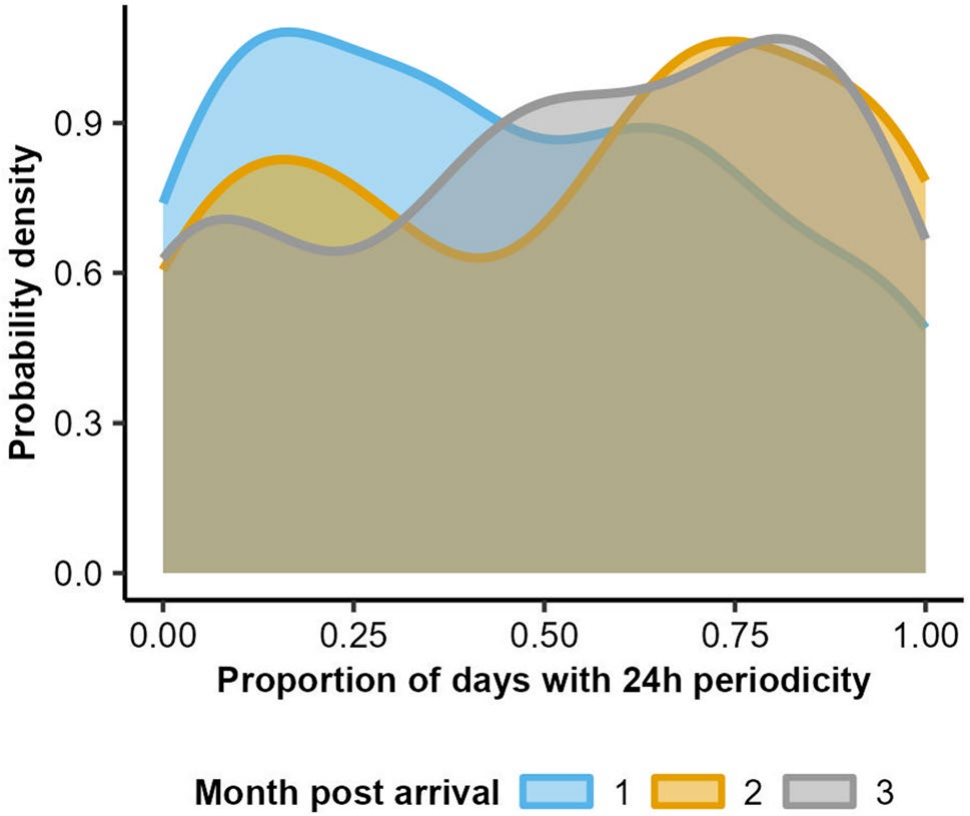
Probability density plot of the proportion of days on which pigs showed a significant circadian rhythm (median *P*-value between 23.5 and24.5 h < 0.05) in feed intake, split per month after arrival at the growing-finishing farm. Higher probability densities reflect more pigs with that proportion of days with 24 h periodicity.

On average, pigs showed a significant circadian rhythm (i.e. repetition in behaviour at a periodicity of 23.5–24.5 h) in feed intake on 57% ± 3% of days (mean ± standard error) of the growing-finishing phase, ranging from 0 to 100%. The proportion of days upon which pigs showed a circadian rhythm became larger in later months of the growing-finishing phase (Fig. 2). In month 1, most pigs did not show a circadian rhythm in feed intake for the majority of days, while in month 3 most pigs did show a circadian repetition for the majority of days. In month 2, two groups could be distinguished; one showing a circadian rhythm on hardly any days and one on most days of the month.

**Figure 3:**
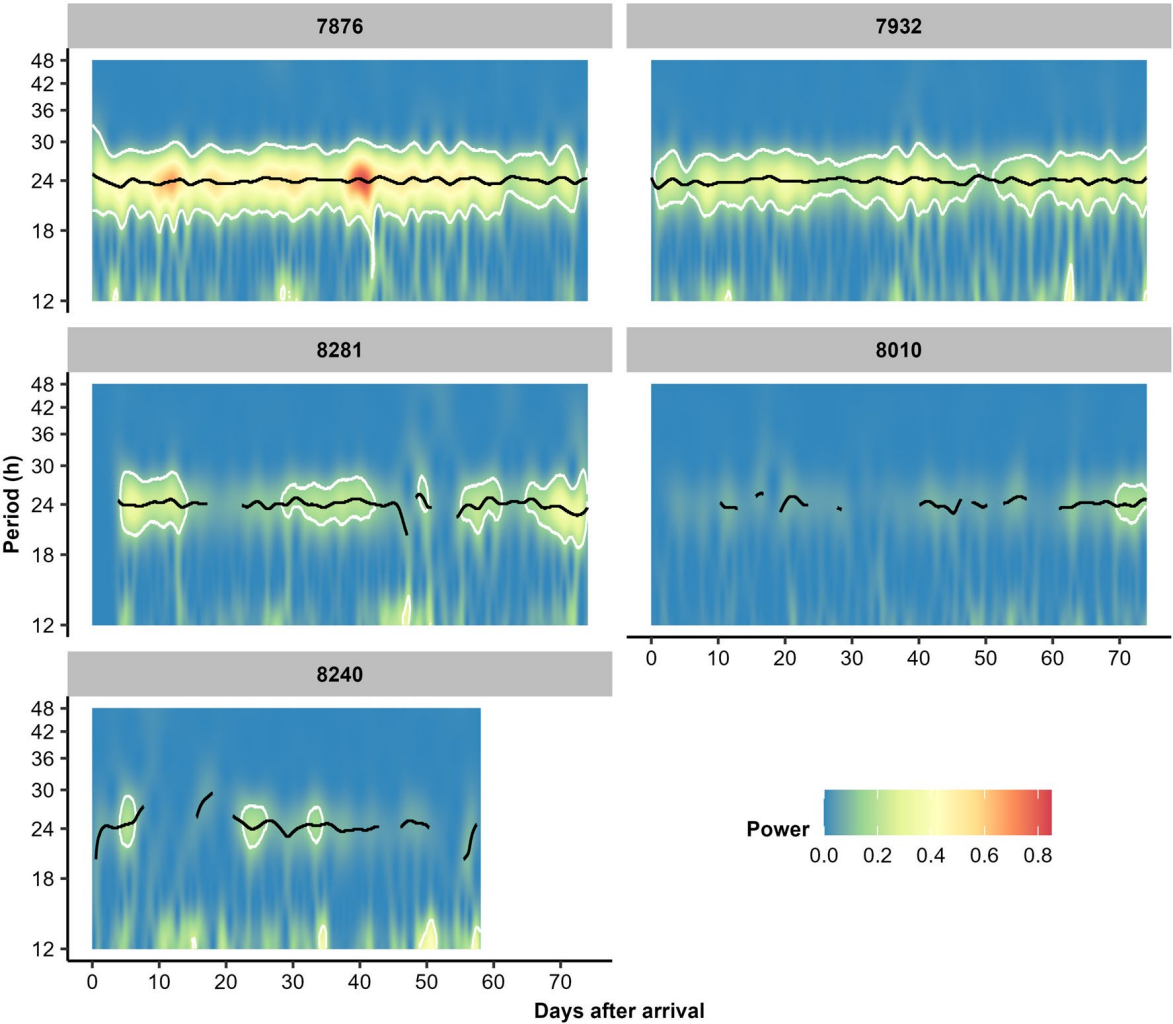
Wavelet spectra of five example pigs (pig numbers given in grey blocks above the plots) during the full growing-finishing phase, visualising detected periodicity (between 12 and 48 h) in feed intake. The colours represent the strength of the periodicity (power), the black line the most likely periodicity and the white lines envelop periodicity that is significantly stronger than white noise (*P* < 0.05), where white noise would reflect random feed intake behaviour. Pig No. 8240 has missing data at the end of the growing-finishing phase due to feeding station malfunctioning.

The concept of rhythmic repetition in individual pig behaviour is further illustrated using example pigs (Fig. 3). Here, the first two pigs (No. 7876 and 7932) show a strong circadian rhythm during the entire growing-finishing phase (illustrated by yellow and red colours within the significance limits), while the last two pigs (No. 8010 and 8240) show hardly any circadian rhythm at all. An intermediate pig (No. 8281) shows a circadian rhythm during certain weeks of the growing-finishing phase but not during others, where more periods with a circadian rhythm were detected in the second half of the growing-finishing phase than in the first half.

### Describing diurnal patterns

Adding diurnal patterns to a GAM with only the across-days trend improved model fit in 100% of pigs, and modelling diurnal patterns for all 6 periods improved model fit over using only 1 diurnal period in 78.6% of the pigs. This warrants the further use of the full model with one daily trend and 6 periods of diurnal patterns. With the selection criterion of a minimum of 7 days available per pig per period, 86 of the total 98 pigs were included for analysis in period 1 (d5–18), 94 in period 2 (d19–32), 95 in period 3 (d33–46), 97 in period 4 (d47–60), 74 in period 5 (d61–74), and 52 in period 6 (d75–83). As period 6 contained only little more than half of the pigs in the total dataset, these results were not considered representative of all the pigs and are therefore not considered further. GAM feature “number of peaks” appeared to have very little variation in the data (for the full data (i.e. per pig period), mean ± standard error: 2.3 ± 0.0; range: 1–4; 80% of observations had 2 or 3 peaks), hence this parameter could not be included in further analysis.

Means and standard errors of each feature for each period are presented in Table 1, along with the effects of period. There was an effect of period, indicating a trend through time, on the timing (*χ*^2^ = 97, *P* < 0.01), height (*χ*^2^ = 51, *P* < 0.01), and width of the highest intake peak (*χ*^2^ = 15, *P* = 0.01), the lowest intake (*χ*^2^ = 53, *P* < 0.01), and the lowest (*χ*^2^ = 42, *P* < 0.01) and highest probabilities to start eating (*χ*^2^ = 44, *P* < 0.01), but not on the proportion of intake obtained at night (*χ*^2^ = 5, *P* = 0.25). The highest intake peak occurred earlier in periods 2 and 3 than in the other periods, and became higher and narrower across the periods. The lowest intake and lowest probability to start eating reduced across the periods, while the highest probability to start eating increased.

**Table 1:** For each period, the mean ± standard error of the features that describe the diurnal intake pattern of included growing-finishing pigs, obtained from the generalised additive models (GAMs).

GAM feature	Period 1	Period 2	Period 3	Period 4	Period 5	*P*-value	ICC	
	Days 5–18	Days 19–32	Days 33–46	Days 47–60	Days 61–74		Pen	Pig
Timing of highest intake peak (Hours post-midnight)	16.4 ± 0.2^a^	14.6 ± 0.2^b^	15.7 ± 0.2^c^	16.0 ± 0.2^ac^	16.3 ± 0.2^a^	< 0.01	0.04	0.39
Highest intake peak (Detrended g/h)	195 ± 5^a^	213 ± 5^b^	228 ± 4^c^	236 ± 5^c^	245 ± 9^c^	< 0.01	0.02	0.10
Width of highest peak (Detrended g/h)	17 ± 2^a^	18 ± 1^ab^	21 ± 1^b^	23 ± 2^b^	24 ± 3^ab^	0.01	0.00	0.06
Lowest intake (Detrended g/h)	41 ± 2^a^	38 ± 2^ab^	35 ± 2^bc^	32 ± 2^cd^	29 ± 2^d^	< 0.01	0.06	0.49
Proportion of intake at night	0.17 ± 0.00	0.17 ± 0.00	0.18 ± 0.01	0.17 ± 0.01	0.17 ± 0.01	0.25	0.12	0.32
Lowest probability to start eating	0.18 ± 0.01^a^	0.17 ± 0.01^bc^	0.17 ± 0.01^ab^	0.15 ± 0.01^cd^	0.14 ± 0.01^d^	< 0.01	0.00	0.53
Highest probability to start eating	0.63 ± 0.02^a^	0.65 ± 0.01^b^	0.68 ± 0.01^bc^	0.69 ± 0.01^cd^	0.72 ± 0.01^d^	< 0.01	0.11	0.44

The *P*-value reflects whether the feature differed between periods, and if so the superscripts (a, b, c and d) show which periods differed. For each feature, the intraclass correlation coefficient (ICC) is given for both pens and pigs, reflecting which part of the variation in the feature is explained by a pen resp. pig effect. Higher values for the ICC indicate more variation explained by the pen or pig effect, meaning that for low ICCs pens/pigs differed more from each other and were more consistent across time than for high ICCs. Note that for the width of the highest peak higher numbers reflect narrower peaks and vice versa.

The ICCs of pen and pig (Table 1) reflect the proportion of variation in the data explained by a pen respectively pig effect—in other words, how consistent a pen or pig is for a feature from period to period while differing between pens or pigs. The pen ICCs were generally weak, ranging from 0 to 0.12, meaning that pens were not consistent in their diurnal pattern features from period to period or differed little from each other. The pig ICCs had a larger range, from 0.06 to 0.53. Moderate ICCs for pig were found for the lowest intake (ρ = 0.49), the lowest probability to start eating (ρ = 0.53), and the highest probability to start eating (ρ = 0.44). This means that pigs were moderately consistent from period to period for these aspects of their diurnal patterns.

**Figure 4:**
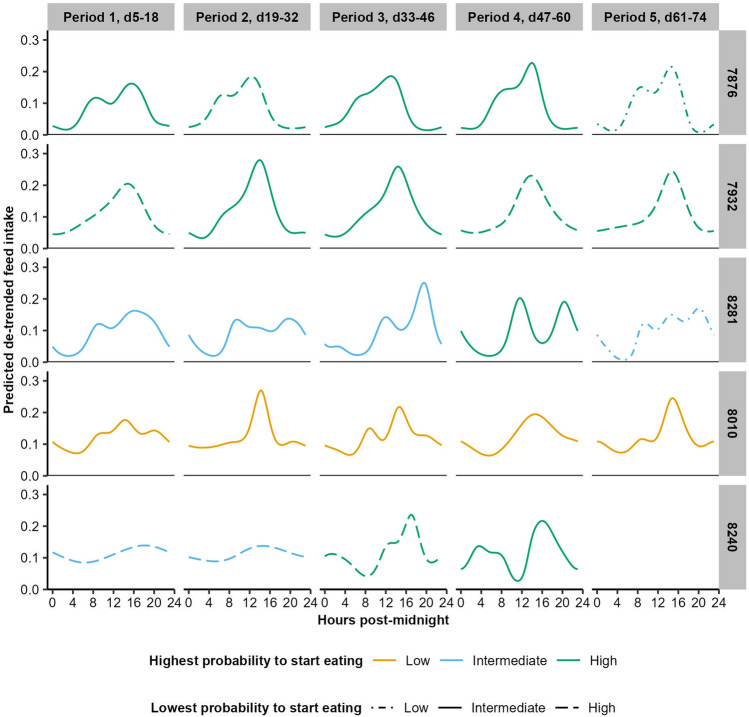
Predicted diurnal intake pattern of five example pigs (pig numbers given in grey blocks on the right) throughout the five periods of the growing-finishing phase. Each period contains at least 7 days of hourly feed intake data; one period of pig No. 8240 did not meet this criterion due to feeding station malfunctioning. The colour and line type reflect the highest and lowest probabilities to start eating as the day-to-day consistency within each period, labelled as low (mean(error) − 0.5 sd(error); lowest probability: ≤ 0.12, highest probability: ≤ 0.62), high (mean(error) + 0.5 sd(error); lowest probability: ≥ 0.20, highest probability: ≥ 0.75), or intermediate (values in between) for illustration purposes.

The differences in diurnal patterns between pigs and periods are further visualised using the same example pigs as for the wavelets (Fig. 4). Pig No. 7876 showed an alternans feed intake pattern in every period of the growing-finishing phase, consistent both across periods and across days within the period. Day-to-day consistency was especially seen for moments on which the pig almost always ate (highest probability to start eating) rather than for moments on which the pig hardly ever ate (lowest probability to start eating). Pig No. 7932 also showed a consistent pattern throughout the periods, but had only an afternoon peak. In addition, this pig was consistent from day-to-day regarding moments on which it almost always ate, but not in moments on which it almost never ate. While these two pigs mainly ate during the day, pig No. 8281 showed feeding activity both during the day and in the evening / beginning of the night, but not later at night. The moment at which it fed is more variable from period to period as well as within the period, with mostly intermediate day-to-day consistency. However, in the final period the pig consistently had a break in feeding at night. Pigs No. 8010 and No. 8240 exemplify feeding activity throughout the night. Pig No. 8010 still concentrated its main intake peaks during the day and ate at lower levels during the morning and night, but the timing of this higher and lower activity was irregular from day to day as the highest probability to start eating was relatively low and the lowest probability to start eating intermediate. Pig No. 8240 showed a clear change with age, where initially the pig ate throughout the day and night—at different moments from day to day—and in later periods (3 and 4) the pig mainly ate during the afternoon, night and very early morning. This pig also became more regular in day-to-day consistency, as in these later periods the pig commonly ate at the same moment from day to day.

### Associations between and within circadian rhythms and diurnal patterns

**Figure 5:**
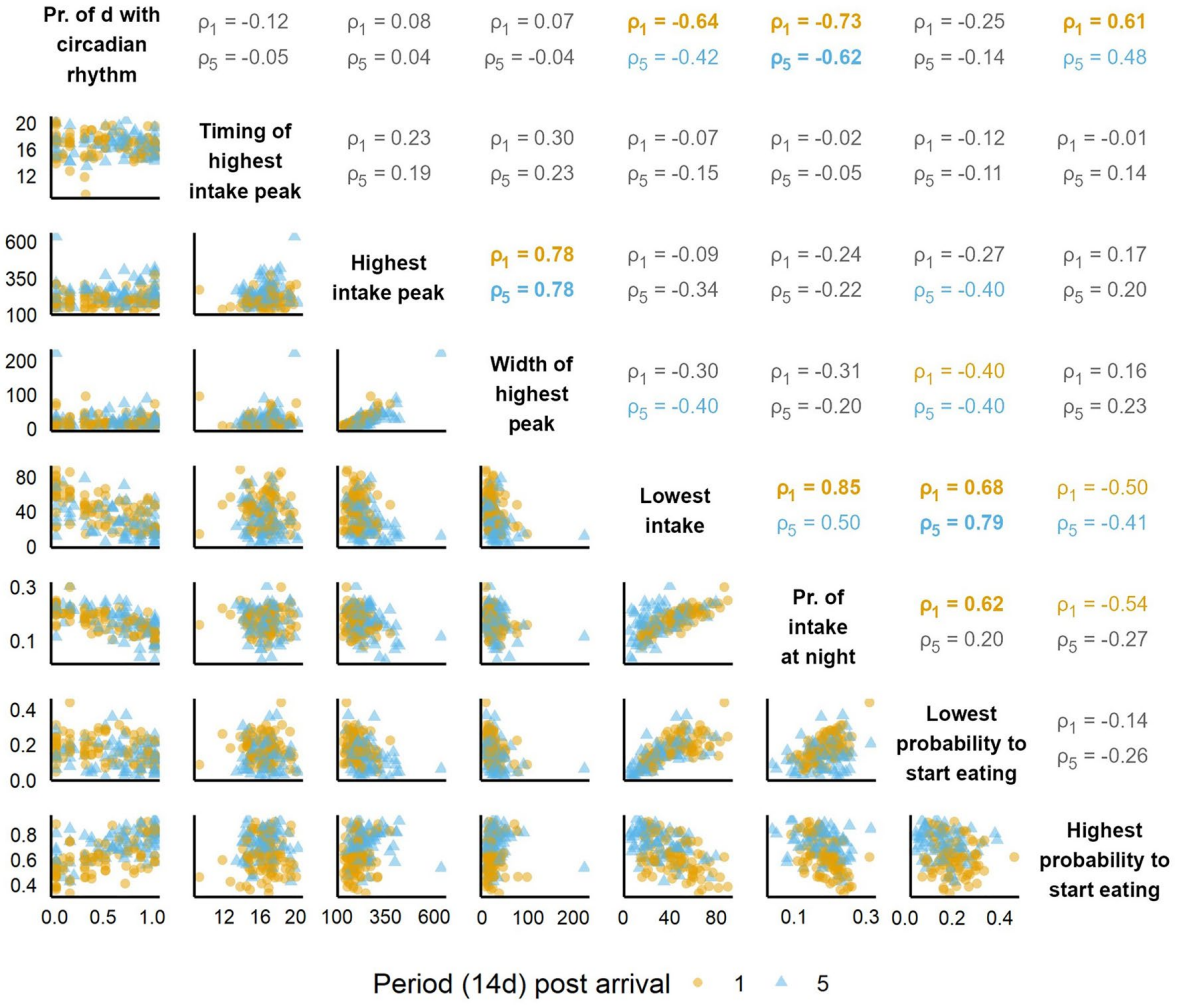
Pair plots (below diagonal) and Spearman correlation coefficients (ρ, above diagonal) between all features of the wavelets (circadian rhythms) and generalised additive models (quantifying diurnal patterns), in periods 1 (yellow circles) and 5 (blue triangles). Correlation coefficients are shown in the colour of the period if moderate or strong (≥ 0.40) and in bold if strong (≥ 0.60). Variables from the wavelets include the proportion of days (Pr. of d) with a circadian rhythm, and all other variables originate from the generalised additive models. Timing of the highest intake peak is expressed in hours post-midnight, and the height and width of the highest intake peak and the lowest intake in de-trended g/h.

Spearman correlations were calculated between all features for each of the five time periods of the growing-finishing phase, the results of which are visualised in the Supplementary Results (Supplementary Fig. S1). As correlations between the periods were mostly similar except for some general trends, only the results of the most extreme periods 1 and 5 are included in detail here. In both periods, correlations ranged from weak to strong, with all features except the timing of the highest intake peak moderately or strongly correlated to at least one other feature. Of the 28 tested pairs, in period 1 three correlations were moderate and seven were strong, with the strongest correlations involving the proportion of days with a circadian rhythm, the height and width of the highest peak, the lowest intake, and the lowest and highest probabilities to start eating. In period 5, only three strong correlations remained, and seven moderate correlations were found. The additional moderate correlations mainly involved the width of the highest peak and the highest probability to start eating, and correlations which were previously strong, including highest probability to start eating and the proportion of days with a circadian rhythm. Scatter plots of all relationships between the features in periods 1 and 5 and their corresponding Spearman rank correlation coefficients are shown in Fig. 5. In period 5, one outlying data point can be seen for which no explanation (e.g. pig disease, poor model fit) is known. Removal of this point from the dataset had only limited effect on analysis results and did not change the conclusions (results not shown), hence it was retained in the dataset.

## Discussion

This study’s objectives were threefold. First, we aimed to explore whether individual pigs show circadian rhythms in feed intake throughout the growing-finishing phase. Second, we aimed to describe what the diurnal patterns in feeding behaviour look like for individual pigs. Finally, we aimed to see how these diurnal patterns changed with age and how consistent groups of pigs and individual pigs were in their diurnal feeding activity.

### Detecting circadian rhythms

Wavelet analysis revealed periodicity in the feed intake of (some) pigs solely on a 24 h basis, hence pigs showed circadian rhythms as opposed to different types of periodicity (e.g. ultradian rhythms). This corresponds to previous studies at group level, which also identified distinct circadian rhythms in the feeding and activity behaviour of pigs^35^. Nevertheless, this circadian rhythm could in individual pigs only be identified on an average of 57% of the fattening days, with individual pigs ranging from 0 to 100% of days displaying a circadian rhythm. Indeed, the example pigs demonstrated clearly that pigs ranged from a steady circadian rhythm throughout the growing-finishing phase to hardly any significant rhythm, with in-between pigs displaying periods with a circadian rhythm interspersed by non-rhythmic periods. This contradicts the general consensus that most animals show natural circadian rhythms enforced by internal physiology and external cues (Zeitgebers)^27,28^ and instead reveals substantial individual differences between pigs. One possible explanation could be that the pigs were too young to show strong circadian rhythms, as it is well known in other mammals that the circadian rhythm gradually develops with age^27^. This hypothesis is supported by our finding that the proportion of days with a circadian rhythm was higher in later months of the growing-finishing phase. A previous study found that 10–12 weeks old pigs had circadian rhythms in feeding behaviour at group level^40^, which is at a younger age than most pigs in this study began showing a circadian rhythm on the majority of days (month 2 of the growing-finishing phase, which corresponds to ± 13–18 weeks of age). This difference could be due to the averaging of patterns of different pigs in the group-level study, which would give a more flattened-out and stable pattern from which circadian rhythms may be more evident than when pig rhythms are analysed individually.

Nevertheless, if age is the only explanatory factor it would be expected that pigs would either show a circadian rhythm consistently, slowly develop it across the growing-finishing phase or not show it at all. Instead, the wavelet spectra (see example pigs in Fig. 3) reveal that pigs with intermediate circadian rhythms across the fattening phase showed periods with a circadian rhythm interspersed by periods with no detectable rhythm, which is unlikely to be explained by age development. We propose this pattern may be due to competition at the feeder, unseen welfare issues, or (changing) individual preferences of pigs. Competition at the feeder is well-known to affect pig feeding behaviour^6^, both at daily^32,58^ and diurnal levels^32,33,59^. It is hence likely that the strength of circadian rhythms in feed intake behaviour would be influenced by competition as well. Eleven pigs per feeding space is judged to be around the limit for compromised daily feed intake and is associated with feed competition at peak hours^59,60^. Indeed, although the mean feeder occupation rate was relatively low on average (mean ± standard error of pens, month 1: 41 ± 0%; month 2: 41 ± 0%; month 3: 29 ± 0%), if only the busiest hour was considered occupation rates in this experiment were on average very high (month 1: 93 ± 1%; month 2: 95 ± 2%; month 3: 82 ± 4%). We theorise that dominant pigs, which have an easier time accessing the feeder^61^, are more able to feed according to a distinct circadian rhythm than subordinate pigs, which have to feed when the dominant pigs do not want to and allow other pigs to access the feeder. It could be that pigs show weaker circadian rhythms the lower their social rank becomes, as their behaviour would need to be increasingly flexible to obtain access to the feeder. This hypothesis could be tested by comparing the social ranks of individual pigs (which may also vary over time due to for example health issues) to the strength of their circadian rhythm. An alternative hypothesis could be that pigs may show phases without circadian rhythms in periods of stress or welfare issues, similar to deviations from day-level basal feeding behaviour in such periods^62^. We attempted to exclude days surrounding welfare issues, but some welfare issues may have gone unnoticed (e.g. subclinical disease, social disturbances, hierarchy changes). Nevertheless, unnoticed welfare issues may seem unlikely to explain the complete lack of circadian rhythms throughout the growing-finishing phase in some pigs, as it would imply that many pigs suffered from severe enough yet unnoticed welfare issues to disturb their behaviour throughout the entire growing-finishing phase. It is known in humans, though, that clinical depression and anxiety are linked to disturbances in the circadian rhythm^63,64^. This could suggest that longer-term negative mental states may hamper (the development of) circadian rhythms in growing-finishing pigs as well, especially as pigs are housed in barren environments and are subject to feeder competition, both of which could result in long-term negative states. A final theory could be that the large differences between pigs are simply due to individual preferences and differences, which could link to behaviour as well as physiology. Modelling studies have previously indicated the involvement of physiological processes, such as hormone cycles and digestion^65^, as well as behavioural strategies during feed competition^60^ in the diurnal feeding patterns of pigs. In addition, pig personality has been proposed to influence day-level feeding behaviour by authors of empirical studies^66,67^. These findings could possibly extrapolate to the strength of circadian rhythms in feed intake as well.

### Describing diurnal patterns

The shape of individual diurnal patterns throughout the growing-finishing phase was, in general, a two-peak pattern with the largest peak in the afternoon. This pattern corresponds with the well-known alternans pattern that is seen when feed intake is averaged at group level or across days^30,68^. With age, this alternans pattern became more distinct as the afternoon peak became higher and narrower while the period of fasting at night enlarged—albeit without leading to a smaller proportion of intake obtained at night. Increasing feeding rate with age^69^ may explain the narrowing of time frames with feed intake, as it would allow pigs to have similar intake in less time. The temporary shift of the main feeding peak from later to earlier in the afternoon at the end of the growing phase (periods 2 and 3, Table 1) does not fit in this development pattern, but may be due to a few outlier pigs whose morning intake peak was slightly larger in those periods than their afternoon intake peak, dragging the average down. This demonstrates that the timing of the highest intake peak is sensitive to outliers and may, as calculated now, not be the most reliable feature of diurnal patterns. The increasing day-to-day consistency, indicated by an increasing highest probability and decreasing lowest probability to start eating, suggests a strengthening of individual diurnal patterns with age. These quantitative results correspond to visual results of previous studies at group level, where the alternans pattern in graphs was more easily recognisable in later than earlier weeks of the fattening phase^70,71^.

Although the alternans pattern is seen in general, the individual plots, scatter plots and ICC scores suggest that the expression of this pattern differs between pigs (i.e. a range of other diurnal patterns are shown). In addition, the relatively moderate highest and lowest probabilities of a pig eating at the same moment on each day suggest that diurnal intake patterns differ from day to day and are not always well reflected by the modelled period pattern. Yet, the probabilities of a pig eating at a certain moment reached as high as 99%, indicating that at least some pigs were very consistent in their diurnal feed intake from day to day. It thus seems that both the type of pattern shown and how consistently this pattern is shown from day to day differs between pigs. Especially for patterns with relatively flat lines (e.g. Fig. 4, pig No. 8240, first 2 periods) it seems logical that pigs are unlikely to have adhered such a flat-line pattern (i.e. ate similar quantities every hour). Instead, it is more likely that they ate very frequently but at slightly different moments from day to day (e.g. every two or three hours), leading to a flat line as the 14d-average.

Besides from day to day, there were also differences in the development of diurnal patterns across the growing-finishing phase (i.e. across the five periods). Little of this variation could be explained by pen effects, as the pen-level ICCs were low, which suggests that there is little consistent difference in diurnal feed intake patterns between pens. This could be explained by the use of a single-space feeder, which generally has such high occupation levels that at pen level the diurnal intake pattern is always similar. As only one pig can feed at any point in time, there is only a limited range of behavioural strategies that pigs could apply within the feeder occupation levels, giving similar pen-level averages. Consequently, the within-pen—or between-pig—variation should be larger than the between-pen variation. Indeed, at pig level, the ICCs ranged across the features from weak to moderate, meaning that at least for some features the period variation could be partially explained by pig effects. More practically, this means that for some features of diurnal intake patterns pigs were quite consistent from period to period, while varying between each other. Pig consistency across periods was especially evident for the lowest intake and lowest and highest probabilities to start eating, suggesting that individual pigs are relatively consistent across the growing-finishing phase in the extent to which they have a fasting period at night and feed similarly from day to day. These differences between pigs in day-to-day consistency and night feeding across periods can be clearly seen in the example pigs as well, where some pigs did not eat at night in any period and others in every period. As for circadian rhythms, it could be that this diversity in feeding behaviour is due to competition at the feeder, which forces subordinate pigs to eat more at night^32,33^. Additionally, hormone cycles^65^ and pig personality (e.g. dominance, general activity, diurnal activity preference)^60,66,67^ could be involved.

### Associations between and within circadian rhythms and diurnal patterns

The existence of different types of diurnal patterns in feed intake is further corroborated by the correlations between the different features of circadian rhythms and diurnal patterns. The strong correlation between the height and width of the highest peak suggests that pigs either ate more concentrated at a specific time of day (high and narrow peaks) or more spread-out across the day (low & wide peaks). This spread-out pattern could in some cases be representative of inconsistent eating from day-to-day, leading to the flat-line pattern discussed previously, however this likely only explains part of the peak width and height as those only moderately correlated with the lowest probability to start eating. Alternatively, the correlation between peak width and height could be related to the well-documented distinction between meal eaters and nibblers^29,67^, a possible relationship that could be tested by comparing peak height and width to the daily feeding frequency and intake per visit or meal. The correlations between the proportion of days with a circadian rhythm, lowest intake, proportion of intake obtained at night and highest probability to start eating suggest that pigs with clear circadian rhythms are those that have similar patterns between consecutive days and do not feed at night, while pigs with no clear circadian rhythms feed more at night and differ in their diurnal intake pattern from day to day. It could again be that this relates to pigs’ social ranks (as previously discussed), or it could be that the wavelet analysis method was better able to pick up circadian rhythms if there was a clear gap between intake activity compared to when the hourly differences were more subtle. The latter theory seems, however, unlikely as in that case a negative correlation between the proportion of days with a circadian rhythm and the lowest probability to start eating would have been expected, but this was not observed. Although the correlations sustained across periods, those between circadian rhythms and features related to night feeding were weaker in period five compared to period one. This suggests that all or at least a wider range of types of diurnal patterns developed into a circadian rhythm by then as well, which is in line with the idea of a general development of circadian rhythms with pig age, also for pigs that do not show a clear fasting period at night.

Interestingly, the two measures of day-to-day consistency—the lowest and highest probabilities to start eating—were not correlated with each other (Period 1: ρ = − 0.14; Period 5: ρ = − 0.26). Visualisations of period diurnal patterns of individual pigs (Fig. 4) indeed showed that pig periods with high values for the highest probability to start eating, which would indicate high day-to-day consistency, did not necessarily have low values for the lowest probability to start eating, which would also indicate high consistency. Instead, the two appear to indicate two different aspects of day-to-day consistency, where the highest probability indicates whether a pig shows a clear and consistent peak in its highest feeding activity and the lowest probability whether a pig consistently fasts at some moment during the day (often at night). It could be theorised that, just like feeding behaviour itself (intake, frequency, duration, etc.), consistency in feeding behaviour can be expressed along multiple dimensions. In this study, we have only looked at consistency in the timing of feeding, and then only at the extremes (i.e. highest and lowest probabilities to start eating, with ‘eating’ as a 0–1 measure based on hourly intake). Other dimensions could be considered, such as consistency in the quantity of feed intake at a certain moment of the day, or in diurnal consistency of other feeding parameters such as feeding frequency or rate.

### Study limitations and suggestions for future research

The findings in this study contribute to our understanding of the individual and temporal variation in the diurnal feeding behaviour of growing-finishing pigs within its specific settings (i.e. one pig round in one fattening farm). It should be noted that the feeding behaviour of pigs at the daily level is known to be influenced by many environmental and pig factors, such as feed(er) type^72^, lighting regime^40^, season^73^, pig gender^74^ and breed^67,75^, an influence that is likely to extrapolate to diurnal feeding behaviour. Future research should explore to what extent such environmental and pig factors influence the individual and temporal differences in pig circadian rhythms and diurnal patterns in feed intake. As no clear pen differences were seen, we hypothesise from our results that gender effects may be minimal when pigs are kept in single-sex pens. It would be especially interesting to study group and individual differences in diurnal rhythms and patterns in environments of varying competitive levels (e.g. changing group size, using multi-space feeders at different locations in the pen, or providing a protective crate), during heat stress and during clinical disease, as these are all highly relevant for pig welfare. Pigs of different performance levels (e.g. growth rate, feed efficiency) could be compared on their diurnal feeding behaviour and consistency, and it could be studied whether pigs within the same pen show similar (e.g. all pigs either a strong, interspersed or no circadian rhythm) or complementary (e.g. a mix of pigs with a strong, interspersed or no circadian rhythm) diurnal patterns or development of circadian rhythms to each other. These comparisons could be based on hourly feed intake but also on one of the other main feeding parameters (duration, frequency and rate), and it would be interesting to include parameters on different dimensions of day-to-day and across-period consistency. If relationships with certain features of diurnal intake patterns are found, these could be used to develop an algorithm that automatically detects welfare issues through detection of certain types of features or deviations in features from their basal levels. To be able to detect more subtle, welfare-indicative deviations in these features, it is important to select the features that have a high day-to-day and across-period consistency, yet are sensitive to welfare issues.

## Conclusion

We conclude that growing-finishing pigs can show a circadian rhythm in feed intake, especially at older ages, but that there was a wide range in the extent to which individual pigs adhered to a circadian rhythm. The group-level diurnal pattern in feed intake was alternans, which, similarly to the circadian rhythm, became clearer with age. Nevertheless, the alternans pattern was only displayed by some of the individual pigs while others displayed a range of other patterns. Individual pigs were relatively consistent throughout the growing-finishing phase in whether they showed a fasting period at night and fed at a specific moment of every day. Consistency from day to day also related to the strength of circadian rhythms, where at younger ages stronger rhythms were mainly seen in pigs with a fasting period at night but at later ages circadian rhythms were seen for other patterns as well. In addition, consistency in feeding behaviour appears to have multiple dimensions that are not necessarily correlated to each other, suggesting that several parameters may be required to determine whether a pig behaves consistently or not. We theorise that differences in diurnal feed intake between pigs and over time may be related to development with age, social rank and individual pig preferences or personality. In future research, it would be valuable to explore how features of diurnal feeding behaviour are influenced by pig and environmental factors, especially those related to welfare, such as social stress, thermal stress and clinical disease. This could help identify certain types of diurnal patterns that are indicative of welfare issues, or features of circadian patterns in which pigs are time-consistent and from which more subtle welfare-indicative deviations could be isolated. This knowledge is essential in developing an algorithm that could validly monitor pig welfare from continuous sensor data.

### Supplementary Information

Supplementary Information.

## Data Availability

The datasets generated and/or analysed during the current study as well as code for processing and analyses are available from the corresponding author on reasonable request.
