# Supplementary material for: Circadian rhythms and diurnal patterns in the feed intake behaviour of growing-finishing pigs
Source: Sci Rep. 2023 Sep 25;13:16021. doi: 10.1038/s41598-023-42612-1 (PMC10519948; doi:10.1038/s41598-023-42612-1)
Supplement: Supplementary file 1 — Supplementary Information. [file 41598_2023_42612_MOESM1_ESM.pdf]

Supplementary Methods - Data Processing and Cleaning

Jacinta D. Bus[1], Iris J.M.M. Boumans[1], Jasper Engel[2], Dennis E. te Beest[2], Laura E. Webb[1], Eddie A.M. Bokkers[1*]

[1] Animal Production Systems group, Wageningen University & Research, PO Box 338, AH 6700 Wageningen, the Netherlands

[2] Biometris, Wageningen University & Research, PO Box 16, 6700 AA Wageningen, The Netherlands

[*] *Corresponding author: eddie.bokkers@wur.nl*

1. Feeding station data processing and cleaning

All data processing and analyses were performed with R, version 4.1.2[1].

Electronic feeding station records provide the time stamp and trough weight upon the start and end of every feeding visit. These were used to calculate the intake (kg) and duration (s) of the visit, as well as the feeding rate (intake/duration*1000, g/s) and the interval between successive visits of an individual pig (s).

Feeding station data are known to contain errors and thus cleaning of the data is required before use. We used a cleaning algorithm adapted from that developed by Casey et al.[2] and Eissen et al.[3]. This algorithm provides thresholds to identify putative outliers based on 1) intake, 2) duration, 3) rate, 4) weight differences of the feed in the trough between successive visits, and 5) time stamp overlaps between successive visits which would suggest two pigs were feeding simultaneously. Additionally, it removed 6) days upon which too many visits were removed, to reduce the introduction of bias during data aggregation. Feeding visits exceeding these thresholds were removed from the dataset, or, if only the load cell was affected, had intake and rate removed. In addition, the feeder also registered feeder filling events and ghost visits - when the load cell detected a weight change but no tag was identified by the RFID antenna -, which were removed from the dataset. As one ear tag broke, 12d of that pig's data were missing until a new tag could be attached, during which the pig could feed normally. Finally, due to technical issues, all data from one station from d63 onwards had to be removed. A detailed overview of the applied algorithm, how many visits were removed from the dataset, and for which reason they were removed, is presented in Supplementary Table S1.

Supplementary Table S1 – Steps taken in the cleaning of the feeding data. Specification of the rules used in the algorithm to clean the data, the number and percentage of visits removed due to every rule, and the number and proportion of visits removed and retained after the full cleaning process. If a rule was only applied on a subset of the data, this is specified underneath 'subset'. Visits could be either fully (column 'Do': 'R') or partially ('P') removed, the latter only removing visit intake and rate caused by load cell error. Intake (INT) is given in kg, rate (RAT) in g/s, duration (DUR) in s, following (FWD) and leading (LWD) weight differences in kg, and following (FTD) and leading (LTD) time differences in s.

Step	Label	Subset	Rule	Do	No. visits	% visits
				Size of full dataset	***219,521***	***100***
1	Wrong station		Tag number not in pen	R	34	0.02
2	Ghost visit		Pig = "0"	R	3236[1 2]	1.47
3	Wrong intake		INT < 0.02 or INT > 2.00	R	2496	1.14
4		DUR = 0	INT > 0.01 or INT <-0.01	R	0	0
5	Wrong duration		DUR < 0 or DUR > 3600	R	0	0
6	Wrong rate	INT > 0 & INT < 0.05	RAT > 8.3	R	145	0.07
7		INT = 0.05 & preceded or followed by INT = 0.02	RAT = 1.8	P	663	0.30
8		INT = 0.05 & not preceded or followed by INT = 0.02	RAT = 2.8	P	156	0.07
9		RAT = 0	DUR > 500	R	37	0.01
10		RAT ≠ 0	RAT < 0.03 and RAT > -0.03	R	11	0.00
11	Following and leading weight differences		-0.5 < FWD > 0.65 and 0.95 < FWD > 1.25	P	921	0.42
12			-0.5 < LWD > 0.65 and 0.95 < LWD > 1.25	P	892[3]	0.41
13	Following and leading time differences		FTD < 0	R	5	0.00
14			LTD < 0	R	5	0.00
15	Proportion removed visits		>15% visits of a pig day partially removed	P	1303[4]	0.59
			>15% visits of a pig day manipulated	R	2462[5]	1.12
				Sum visits removed	***12,028***	***5.48***
				Sum visits partially removed	***3,921***	***1.79***
				Total visits unmanipulated	***203,572***	***92.73***

[1] *An additional 62 visits were removed for a pig with a broken ear tag*

[2] *An additional 3535 visits were removed due to a malfunctioning antenna of one feeding station (all data from this station, from d63 onwards)*

[3] *Of which 14 visits had been partially removed previously*

[4] *On 121 pig days*

[5] *On 163 pig days*

The cleaned dataset was aggregated into hourly data to be able to assess diurnal patterns. Thus, hourly feed intake was calculated for each pig by summing the intake of all visits that started within that hour. If no visits had occurred, 0kg was registered.

2. Removing putative deviating days from the cleaned feeding data

To be able to study basal patterns, we aimed to remove putative deviating days from the dataset. To achieve this, we firstly removed all days before the first health observations (326 pig days), to eliminate the effect of habituation to the new environment and to avoid inclusion of sick pigs. In addition, only data until the first pigs left to the slaughterhouse was retained, to exclude influences of different group sizes and changing social ranks as much as possible (539 pig days). Finally, health data was used to remove the days surrounding severe health issues. Health issues were identified via twice weekly farm visits at pig level, using the scoring described in our larger health observations protocol deposited in the DANS database[4] (lying bumps and conjunctivitis were not yet scored in this experiment, only pig-level observations were considered). Only health issues considered severe were removed, the applied thresholds can be found in Supplementary Table S2 along with the number of days removed for each issue. If a severe health issue was identified, all data from that pig from the 3d before to 3d after diagnosis were removed. Data from one pen were fully excluded due to an ear-biting outbreak shortly after arrival followed by a tail-biting outbreak that lasted until the first slaughter date (757 pig days). The final dataset consisted of 6348 complete days on 98 pigs from 9 pens, with a median of 65 days per pig (range 16-79d).

Supplementary Table S2 – Quantification of feeding data removal due to health issues. An overview of the number of pig days removed from the dataset due to severe health issues. For each health issue, the range of scores and the threshold above which an issue was considered 'severe' is shown (≥), followed by the number of observation days on which the severe issue was seen, how many pigs had days removed because of it, and how many pig days were removed because of it (count). In addition, the mean, minimum and maximum number of days individual pigs had removed for this issue is shown. The totals reflect the total number of observation days removed, pigs affected (also in % of all pigs), pig days removed (also in % of all pig days) and mean, minimum and maximum number of days removed per pig. These totals may differ from the sums as (pig) days could be removed due to more than one health issue.

**Health issue**	**Range**	**Threshold**	**Days observed**	**Pigs affected**	**Days removed**			
					Count	*Mean*	*Min*	*Max*
Ear damage	0-4[1]	2	63[3]	31[3]	439	14.2	7	56
Tail damage	0-5	2	42[3]	25[3]	278	11.1	5	33
Flank damage	0-2	1	22	20	150	7.5	5	14
Lameness	0-3	2	19	15	127	8.5	5	19
Shivering	0-1	1	9	3	59	19.7	14	26
Bursitis	0-2	1	6	5	42	8.4	7	14
Skin lesions	0-3[2]	3	5	5	35	7	7	7
Pumping	0-1	1	1	1	7	7	7	7
Body condition	0-2	1	0					
Hernia	0-2	1	0					
Panting	0-1	1	0					
Rectal prolapse	0-2	2	0					
Skin disease	0-2	2	0					
Total			***147***	***68***	***916***	***16.7***	***5***	***56***
Total (%)				***69***	***12.5***			

[1] Highest score of ear base and tip

[2] Highest score of body parts front, middle or back

[3] Excluding the removal of the full pen with an ear and tail biting outbreak

References

1. R Core Team. R: A language and environment for statistical computing. (2021).

2. Casey, D. S., Stern, H. S. & Dekkers, J. C. M. Identification of errors and factors associated with errors in data from electronic swine feeders. *Journal of Animal Science* **83**, 969–982 (2005).

3. Eissen, J. J., Kanis, E. & Merks, J. W. M. Algorithms for identifying errors in individual feed intake data of growing pigs in group-housing. *Applied Engineering in Agriculture* **14**, 667–673 (1998).

4. Bus, J. D., Walderveen, A. van, Bolhuis, J. E., Boumans, I. J. M. M. & Bokkers, E. A. M. Protocol for health observations in growing-finishing pigs. *DANS* (2023) doi:10.17026/dans-xt2-xpej.

Supplementary Results - Temporal Correlations

Jacinta D. Bus[1], Iris J.M.M. Boumans[1], Jasper Engel[2], Dennis E. te Beest[2], Laura E. Webb[1], Eddie A.M. Bokkers[1*]

[1] Animal Production Systems group, Wageningen University & Research, PO Box 338, 6700AH Wageningen, the Netherlands

[2] Biometris, Wageningen University & Research, PO Box 16, 6700AA Wageningen, The Netherlands

[*] *Corresponding author: eddie.bokkers@wur.nl*

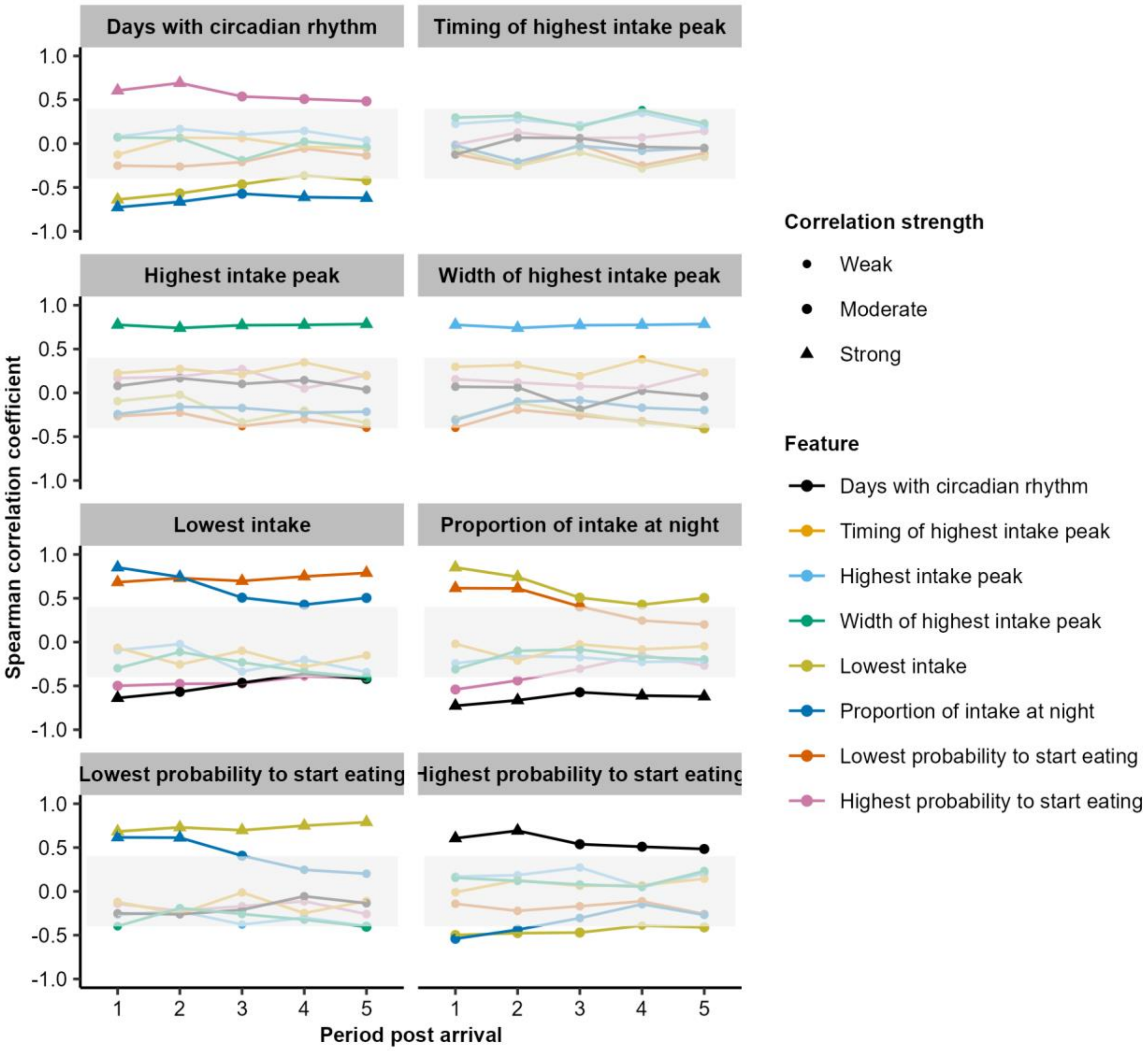

Supplementary Figure S1. Spearman correlation coefficients of features extracted from the wavelet analysis (Proportion of days with circadian rhythm) and generalised additive models (other features), per 14d period of the growing-finishing phase. Weak correlation coefficients (> -0.40 and < 0.40) are greyed out, and strong correlation coefficients (≥ 0.60 or ≤ -0.60) are triangular.
